# High fat-induced inflammation in vascular endothelium can be improved by *Abelmoschus esculentus* and metformin via increasing the expressions of miR-146a and miR-155

**DOI:** 10.1186/s12986-020-00459-7

**Published:** 2020-05-13

**Authors:** Luoning Gou, Geng Liu, Rong Ma, Anita Regmi, Tianshu Zeng, Juan Zheng, Xueyu Zhong, Lulu Chen

**Affiliations:** 1 Department of Endocrinology, Union Hospital, Tongji Medical College, Huazhong University of Science and Technology, Wuhan, 430022 China; 2 Department of Endocrinology, Tongji Hospital, Tongji Medical College, Huazhong University of Science and Technology, Wuhan, 430030 China; 3 Hubei provincial Clinical Research Center for Diabetes and Metabolic Disorders, Wuhan, China

**Keywords:** Obesity, Endothelium, Inflammation, microRNA, *Abelmoschus esculentus*, Metformin

## Abstract

**Background:**

Obesity is associated with chronic inflammation, which contributes to cardiovascular diseases. MicroRNAs (miRNAs) are reported to be involved in vascular inflammation and atherosclerosis. *Abelmoschus esculentus* (AE) and metformin have been suggested to improve inflammation in vascular system. The aim of this study is to evaluate whether miRNAs are involved in high fat induced endothelial inflammation, and whether AE and metformin improve endothelial inflammation by regulating miRNAs.

**Methods:**

We established high fat treated rats and human aortic endothelial cells (HAECs). AE and metformin were added to explore their effects on endothelial inflammation induced by high fat and the possible mechanism.

**Results:**

The vascular inflammatory genes were increased in rats treated with high fat diet. The decreased miR-146a and miR-155 were involved in endothelial inflammation induced by high fat through targeting IL-1 receptor-associated kinase 1 (IRAK1), TNF receptor-associated factor 6 (TRAF6) and nuclear factor-κB p65 (NF-κB p65), respectively. While AE and metformin could ameliorate the endothelial inflammation by increasing miR-146a and miR-155.

**Conclusions:**

These results indicate that miR-146a and miR-155 play roles in the high fat induced endothelial inflammation, which could be potential therapeutic targets. AE and metformin can attenuate endothelial inflammation through regulating miR-146a and miR-155.

## Introduction

Obesity has emerged as a global epidemic with increased risk of type 2 diabetes and cardiovascular diseases, such as atherosclerosis, stroke, hypertension [1]. It has been commonly assumed that obesity induces a state of chronic inflammation, which potentially contributes to cardiovascular diseases [2]. Endothelium exhibits a critical role in the pathogenesis of obesity-related vascular diseases [3]. However, the pathogenesis of inflammation leading to endothelial dysfunction remains obscure, and becomes a huge challenge in obesity-related diseases.

MicroRNAs (miRNAs) are small, highly conserved noncoding RNAs, which inhibit target gene expression at the post-transcriptional levels. miRNAs mainly bind to the 3′-untranslated regions of specific mRNAs to induce mRNA degradation or translation repression [4]. It has been reported that miR-146a, miR-155, miR-21 are involved in regulating inflammation in vascular endothelium [4–7]. In addition, IL-1 receptor-associated kinase 1 (IRAK1) and TNF receptor-associated factor 6 (TRAF6) have been identified as the direct targets of miR-146a [8, 9], and miR-155 could directly bind to nuclear factor-κB p65 (NF-κB p65) [10]. All these target genes are important mediators of inflammation. Nonetheless, whether these miRNAs as well as their target genes participate in high fat-induced endothelial injury is not fully understood.

*Abelmoschus esculentus* (AE), also known as okra, gumbo or lady’s finger, belongs to the mallow family. It’s a vegetable widely grown in tropical and sub-tropical countries. AE is full of nutrients, such as carbohydrate, proteins, minerals, vitamins, fats and large amount of mucilage which contains dietary fibers [11]. It has been reported that AE or its extract can reduce the risk of diabetes, hyperlipidemia, obesity, cancers and depression [12–17]. In addition, it suggested that the extract of AE could attenuate vascular impairment and reduce the levels of inflammatory factors in load-induced fatigued rats [18]. All these results indicate that AE may be involved in the regulation of glucose and lipid metabolism, as well as in inflammation-induced endothelial dysfunction. However, the specific mechanism of vasoprotective effect of AE remains unclear. Metformin is widely used as the first-line oral drug for type 2 diabetes. Growing evidences revealed that metformin exerted anti-inflammatory and improvement of endothelial function in high fat-induced obesity or diabetes [19–24]. But it still needs further investigation that how metformin exhibits the protective role in endothelial dysfunction.

To identify the roles of miRNAs in high fat-induced inflammation, we established the model of high fat treated rats and human aortic endothelial cells (HAECs). Then, we explored whether and how AE and metformin displayed protective effects on endothelial dysfunction induced by high fat.

## Materials and methods

### Preparation of AE powder

AE powder was prepared from fresh AE. The roots of AE vegetable were removed. Then the fresh AE was cleaned and blanched in boiling water for 3 min. It is further dried by hot air in 75°C for 2 h. The dried AE vegetable was firstly shattered by high speed grinder, and then pulverized by airslide disintegrating mill to obtain the AE ultrafine powder.

### Experimental animals

All animal studies were conducted according to the institutional guidelines and approved by Animal Ethics Committee of Huazhong University of Science and Technology. Six-week-old male Sprague Dawley rats (180-200 g) were purchased from Beijing HFK Bioscience Co., Ltd. (Beijing, China). All rats were kept individually in specific pathogen free (SPF) animal houses under 12-h light/ dark cycle with ad libitum access to water in Laboratory Animal Center of Tongji Medical College, Huazhong University of Science and Technology. All rats were received adaptive feeding for 1 week and then randomly divided into two groups: (1) normal chow group (NC, *n* = 15), with a standard chow diet (24% protein, 66% carbohydrates, and 10% fat) throughout the experimental period, provided by the animal center mentioned above; (2) high fat diet (HFD) group (HF, *n* = 60), with high fat diet (20% protein, 20% carbohydrates, and 60% fat, H10060, Beijing HFK Bioscience Co., Ltd., China). After 10 weeks, all rats in HFD group were further randomly divided into 4 groups and received different treatments: (1) high fat diet group (HF, *n* = 15), rats were kept on with high fat diet for 8 weeks and given equal volumes of 0.9% saline by oral gavage; (2) high-dose AE group (HF-OA, *n* = 15), rats were kept on with high fat diet for 8 weeks and given AE (800 mg/kg) by oral gavage in 0.9% saline vehicle daily for 8 weeks; (3) moderate-dose AE group (HF-OB, *n* = 15), rats were kept on with high fat diet for 8 weeks and given AE (400 mg/kg) by oral gavage in 0.9% saline vehicle daily for 8 weeks; (4) metformin group (HF-Me, *n* = 15), rats were kept on with high fat diet for 8 weeks and given metformin (200 mg/kg, Sigma-Aldrich) by oral gavage in 0.9% saline vehicle daily for 8 weeks. At the end of 19 weeks, all rats were sacrificed by intraperitoneal administration of sodium pentobarbital (40 mg/kg). The body weight was measured every week. Blood samples were collected by cardiac puncture and aortas were isolated immediately and frozen in liquid nitrogen for subsequent procedures.

### Cell culture and treatment

HAECs (Catalog#6100, ScienCell) were cultured in endothelial cell medium supplemented with 10% fetal bovine serum, 1% endothelial growth factor, 100 IU/mL penicillin and 0.1 mg/mL streptomycin (Catalog#1001, ScienCell). Cells were cultured at 37 °C in a humidified atmosphere containing 5% carbon dioxide, with medium changed every 2 to 3 days. One day before treatment, cells were plated and then assigned into groups as follow: BSA (bovine serum albumin) group (with 0.005 g/ml BSA for 19 h), PA (palmitic acid) group (with 0.3 mM palmitic acid for 19 h [PA]), PA-AE group (with 0.3 mM PA and 150 μg/ml AE for 19 h), and PA-Met group (with 0.3 mM PA and 2 mM metformin for 19 h). PA (P5585, Sigma-Aldrich) was solubilized in absolute ethanol and combined with fatty-acid free bovine serum albumin (BSA, 5%) in 60 °C for 2 h with shaking. The PA-albumin solution was sterilized with 0.22 μm filter (SLGP033RB, Millipore) before treating cells.

### Cell transfection

HAECs were plated 1 day before transfection. The mimics and inhibitors of miR- 146a-5p, miR-155-5p, miR-21-5p, the control mimics and control inhibitors were synthesized by Guangzhou RiboBio Techonologies. Cells were transfected with mimics or control mimics at a final concentration of 50 nM, inhibitors or control inhibitors at a final concentration of 100 nM. The transfection was performed with the riboFECT CP (RiboBio, Guangzhou, China) according to the manufacturer’s instructions.

### Biochemical assays

Blood glucose was measured using a glucometer (One Touch Ultra, Lifescan, Milpitas, CA). Serum levels of total cholesterol (TC), triacylglycerol (TG), low density lipoprotein cholesterol (LDL-C), and high density lipoprotein cholesterol (HDL-C) were determined by colorimetric methods according to the manufacturer’s instructions (Nanjing Jiancheng Biotech, Inc., Nanjing, China).

### RNA isolation and quantitative real-time PCR

Total RNA from aortas and HAECs was isolated with RNAiso Plus (9108, Takara, Japan). cDNA was synthesized with the PrimeScript™ RT Master Mix (RR036A and RR037A, TaKaRa, Japan) by using 500 ng of RNA. The RT-PCR analyses were performed using SYBR® Premix Ex Taq™ (RR420L, TaKaRa, Japan) at Roche LightCycler 480II machine (Roche, Mannheim, Germany). Nucleolar small RNA U6 and β-actin were detected as normalizing control to quantify the expressions of miRNAs and other genes. All primers of miRNAs and U6 were designed and synthesized by RiboBio, and other primers were synthesized by TsingKe (Beijing, China). The sequences of primers used are displayed in Supplemental Table 1. Relative gene expression was determined using 2^-ΔΔCT^ method.

### Western blotting

Total proteins from aortas and HAECs were extracted using RIPA containing protease inhibitors (Beyotime, Jiangsu, China). The proteins were separated by SDS-PAGE and transferred to 0.45-um polyvinylidene fluoride membranes (Millipore, Boston, MA). Then the membranes were blocked with 5% non-fat milk and incubated with the primary antibodies overnight at 4 °C. Then the membranes were incubated with HRP-conjugated secondary antibody (Google, wuhan, China), followed by chemiluminescent detection. The primary antibodies used in this study are listed in Supplemental Table 2.

### Statistical analysis

All data were presented as mean ± SEM of at least 3 independent experiments. Statistical comparison was made by one-way ANOVA or Student’s *t-test*, and was performed with IBM SPSS Statistics 19.0. (SPSS Inc., Chicago, IL, USA). *P*<0.05 were considered statistically significant.

## Results

### miRNAs may be involved in high fat induced endothelial inflammation

Compared with normal chow diet, rats fed with high fat diet for 10 weeks showed significant increase in body weight. Consistent with the body weight gain, significant increase in both subcutaneous fat and visceral fat mass was observed in HF group. The fasting glucose was also impaired when rats challenged with high fat diet. Additionally, the lipid profile in serum was also determined. As expected, higher levels of TC, TG and LDL-C as well as lower HDL-C were observed in HF group (Supplemental Figure 1).

**Fig. 1:**
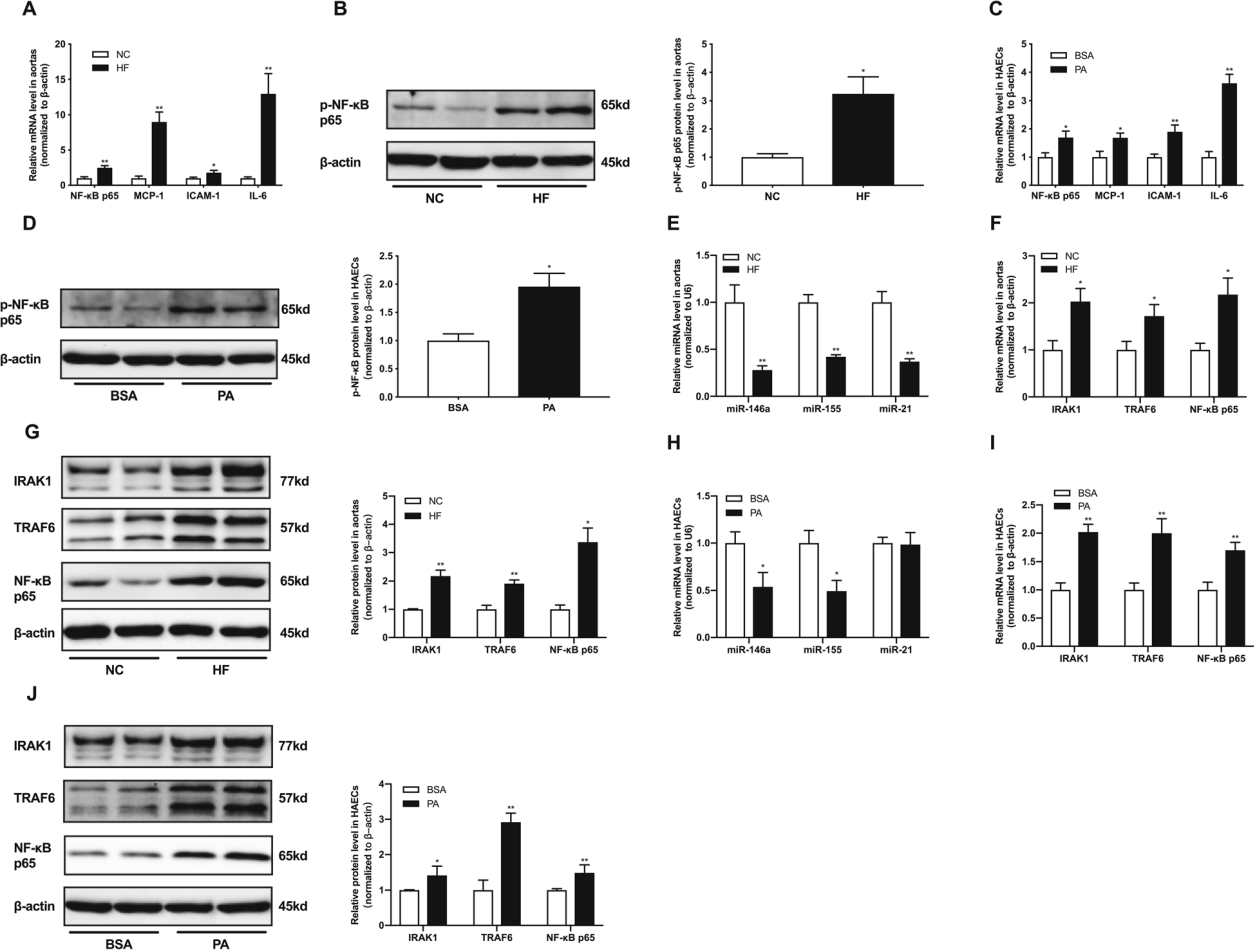
High fat induced endothelial inflammation and miRNAs may be involved in this response. **a**, relative mRNA levels of NF-κB p65, MCP-1, ICAM-1 and IL-6 in rat aortas. **b**, relative protein levels of p-NF-κB p65 in rat aortas. **c**, relative mRNA levels of NF-κB p65, MCP-1, ICAM-1 and IL-6 in HAECs. **d**, relative protein levels of p-NF-κB p65 in HAECs. **e**, relative expression levels of miR-146a, miR-155 and miR-21 in rat aortas. **f**, relative mRNA levels of IRAK1, TRAF6 and NF-κB p65 in rat aortas. **g**, relative protein levels of IRAK1, TRAF6 and NF-κB p65 in rat aortas. **h**, relative expression levels of miR-146a, miR-155 and miR-21 in HAECs. **i**, relative mRNA levels of IRAK1, TRAF6 and NF-κB p65 in HAECs. **j**, relative protein levels of IRAK1, TRAF6 and NF-κB p65 in HAECs. Data are presented as mean ± SEM. ^*^P < 0.05 versus NC or BSA group. ^**^P < 0.01 versus NC or BSA group. *n* = 6–8 rats per group for animal study. *n* = 5 per group for HAECs study

As we all known, fat deposition was always accompanied by chronic low-grade inflammation. We assessed inflammatory factors in aortas of rats, including NF-κB p65, monocyte chemotactic protein 1 (MCP-1), intercellular cell adhesion molecule-1 (ICAM-1) and interleukin 6 (IL-6). These inflammatory factors significantly increased in aortas of HFD rats (Fig. 1a).The phosphorylation of NF-κB p65, an important indicator for inflammation, was markedly elevated in aortas of HFD group (Fig. 1b). At the same time, the expressions of inflammatory factors and phosphorylation of NF-κB p65 were also up-regulated in HAECs treated with PA (Fig. 1c, d).

Next, we examined the expressions of miRNAs related with inflammation in vivo and in vitro. Compared with NC group, the relative expressions of miR-146a, miR-155, and miR-21 were decreased in aortas of HF group (Fig. 1e). Previously, our group demonstrated that miR-146a could directly target IRAK1 and TRAF6 by luciferase assay [9]. NF-κB p65 have been reported to be the direct targets of miR-155 [10]. As shown in Fig. 1, the transcriptional expressions of IRAK1,TRAF6 and NF-κB p65 were significantly increased when exposure to HFD in aortas of rats (Fig. 1f). Meanwhile, the protein levels of IRAK1, TRAF6 and NF-KB p65 were also up-regulated in HF group (Fig. 1g). In HAECs, exposure to PA significantly reduced the expressions of miR-146a and miR-15 but not miR-21 in HAECs (Fig. 1h), which indicates that miR-21 may not be implicated in the inflammation induced by PA in HAECs. Additionally, the mRNA and protein levels of IRAK1, TRAF6 and NF-κB p65 were both elevated in HAECs treated by PA (Fig. 1i, j). Therefore, we speculate that miRNAs may be involved in endothelial inflammation induced by high fat.

### The endothelial inflammation induced by high fat could be improved by regulating miRNA-146a and miRNA-155

**Fig. 2:**
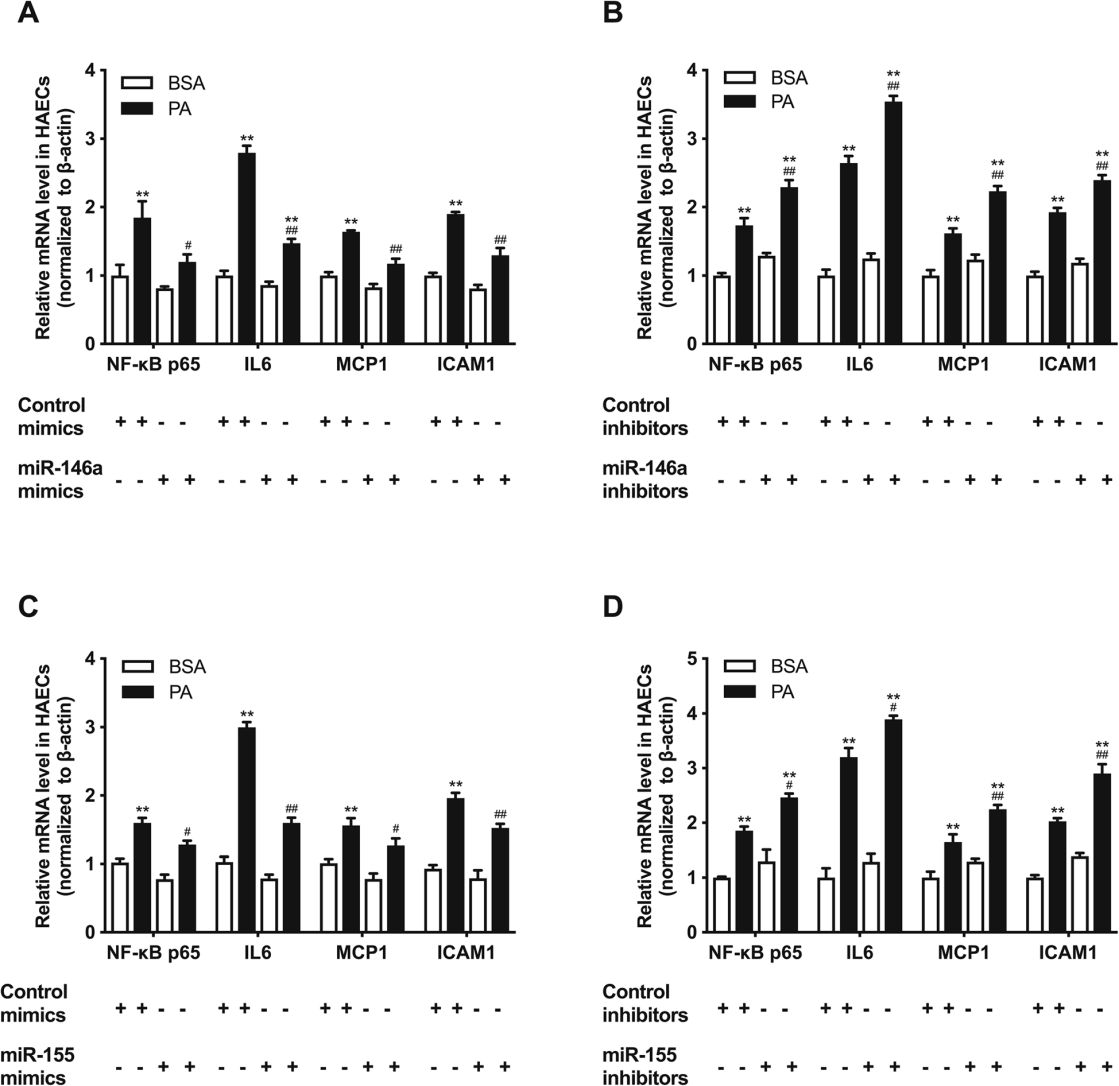
High fat induced endothelial inflammation can be improve by regulating miR-146a and miR-155 in HAECs. **a**-**d**, relative expression levels of inflammatory factors (NF-κB p65, IL-6, MCP-1 and ICAM-1) after transfection with miR-146a mimics, miR-146a inhibitors, miR-155 mimics and miR-155 inhibitors, respectively. Data are presented as mean ± SEM. ^*^P < 0.05 versus BSA group. ^**^P < 0.01 versus BSA group. ^#^*P*<0.05 versus PA group. ^*#*#^*P*<0.01 versus PA group. *n* = 5 per group for HAECs study

To further investigate whether high fat induced endothelial inflammation was mediated by miRNAs, we transfected mimics and inhibitors of miR-146a and miR-155 into HAECs. As shown in Fig. 2, miR-146a and miR-155 mimics could decrease the up-regulation of inflammatory factors (NF-κB p65, IL-6, MCP-1 and ICAM-1) induced by PA in HAECs (Fig. 2a, c). On the contrary, miR-146a inhibitors and miR-155 inhibitors exacerbated the inflammation in HAECs treated with PA (Fig. 2b, d). Regulating miR-21 have no effects on the inflammation in HAECs (Supplemental Figure 2). These results revealed that miR-146a and miR-155 mediated endothelial inflammation induced by high fat and regulating miRNAs can reverse the endothelial inflammation.

### AE and metformin prevent high fat-induced fat accumulation, dyslipidemia and endothelial inflammation

**Fig. 3:**
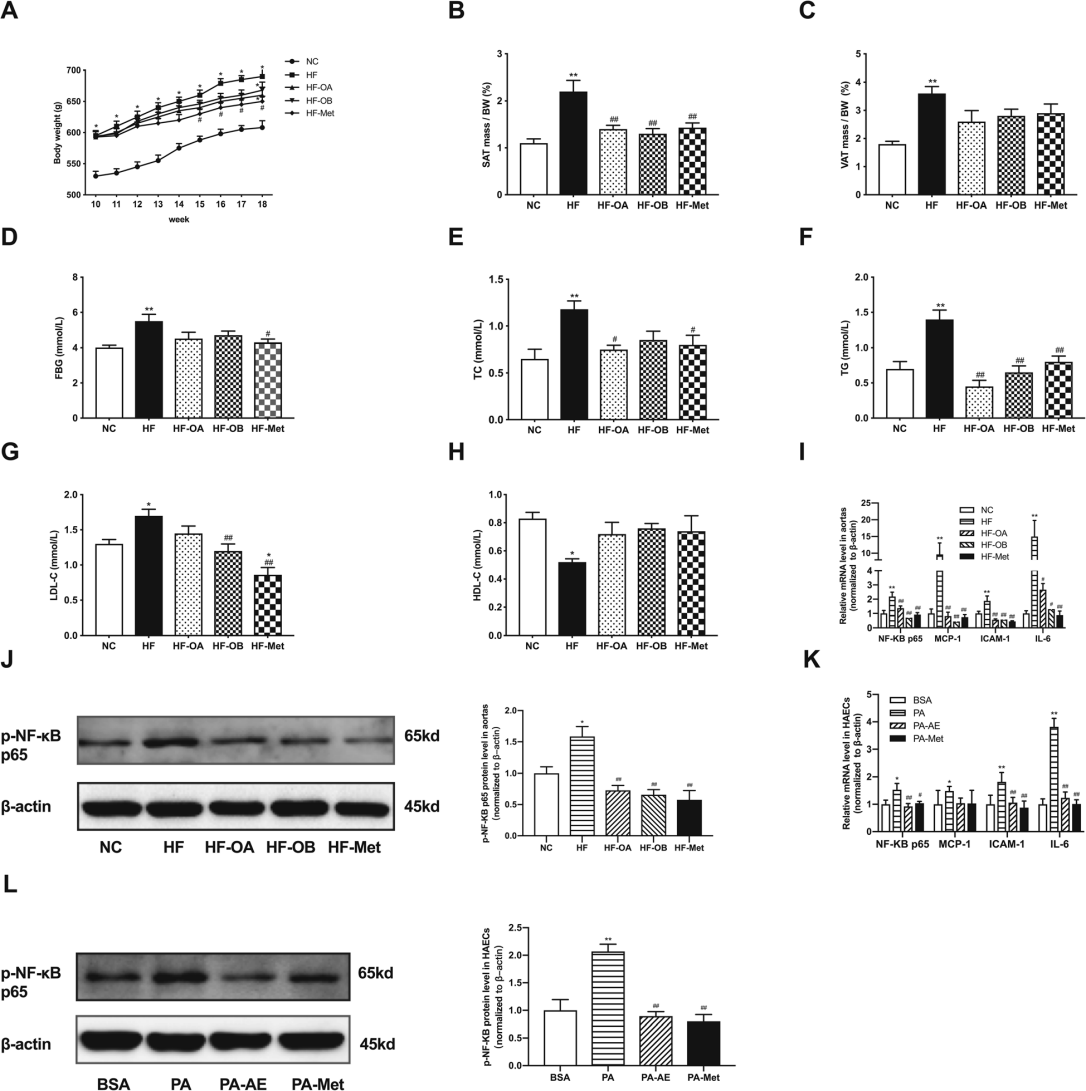
AE and metformin prevent high fat-induced fat accumulation, dyslipidemia and endothelial inflammation. **a**, body weight of rats after treatment with AE or metformin for 8 weeks following high fat diet for 10 weeks. **b**, the percent of subcutaneous fat in rats. **c**, the percent of visceral fat in rats. **d**, the fasting glucose level in rats. **e**-**h**, the TC, TG, LDL-C and HDL-C levels in serum of rats. **i**, relative mRNA levels of NF-κB p65, MCP-1, ICAM-1 and IL-6 in rat aortas. **j**, relative protein levels of p-NF-κB p65 in rat aortas. **k**, relative mRNA levels of NF-κB p65, MCP-1, ICAM-1 and IL-6 in HAECs. **l**, relative protein levels of p-NF-κB p65 in HAECs. Data are presented as mean ± SEM. ^*^P < 0.05 versus NC or BSA group. ^**^P < 0.01 versus NC or BSA group. ^#^*P*<0.05 versus HF or PA group. ^*#*#^*P*<0.01 versus HF or PA group. *n* = 6–15 rats per group for animal study. *n* = 5 per group for HAECs study

To explore the effects of AE and metformin on high fat induced endothelial inflammation both in vivo and in vitro, we fed high fat diet to Sprague Dawley rats for 10 weeks and then treated them with AE/metformin orally for 8 weeks. As shown in Fig. 3a, HFD induced a significant increase in body weight. After treatment with AE and metformin for 8 weeks, the body weight in HF-Met group was decreased compared with HF group (Fig. 3a). AE treatment led to a slight decrease of body weight compared with HF group without significant difference (Fig. 3a). Meanwhile, AE and metformin reduced both subcutaneous fat and visceral fat contents (Fig. 3b, c). AE and metformin could improve the impaired fasting glucose of rats that challenged with high fat diet (Fig. 3d). Additionally, the lipid profile in serum was determined. As expected, higher levels of TC, TG and LDL-C as well as lower HDL-C were observed in HF group, AE and metformin significantly improve the lipid profile (Fig. 3e, f, g and h). The low dose AE (HF-OB) exhibited more efficient in lowering LDL-C level than high dose AE (HF-OA) (Fig. 3g). The effects of different doses of AE and metformin on metabolic parameters were also listed in Table 1.

**Table 1:** Effects of AE and metformin on measured metabolic parameters

Group	BW (g)	FBG (mmol/L)	TC (mmol/L)	TG (mmol/L)	LDL-c (mmol/L)	HDL-c (mmol/L)
NC	608 ± 11.12	3.87 ± 0.5	0.65 ± 0.39	0.70 ± 0.36	1.30 ± 0.39	0.83 ± 0.15
HF	690 ± 12.12^*^	5.50 ± 1.55^**^	1.18 ± 0.42^**^	1.4 ± 0.29^**^	1.70 ± 0.34^*^	0.52 ± 0.21^*^
HF-OA	660 ± 12.94^*^	4.5 ± 1.48	0.75 ± 0.18^#^	0.45 ± 0.32^##^	1.45 ± 0.29	0.72 ± 0.28
HF-OB	668 ± 13.13^*^	4.7 ± 0.77	0.85 ± 0.22	0.65 ± 0.17^##^	1.20 ± 0.23^##^	0.76 ± 0.12
HF-Met	650 ± 8.37^#^	4.3 ± 1.04^#^	0.80 ± 0.34^#^	0.80 ± 0.28^##^	0.86 ± 0.19^*##^	0.74 ± 0.5

*NC* normal chow group, *HF* high fat diet group, *HF-OA* high fat diet and *Abelmoschus esculentus* (800 mg/kg) treatment, *HF-OB* high fat diet and *Abelmoschus esculentus* (400 mg/kg) treatment, *HF-Met* high fat diet and metformin (200 mg/kg) treatment, *BW* body weight, *FBG* fasting blood glucose, *TC* total cholesterol, *TG* total triacylglycerol, *LDL-c* low density lipoprotein cholesterol, *HDL-c* high density lipoprotein cholesterol

Data are presented as mean ± SD. ^*^P < 0.05 versus NC group. ^**^P < 0.01 versus NC group. ^#^*P*<0.05 versus HF group. ^*#*#^*P*<0.01 versus HF group. *n* = 6–15 per group

Besides, treatment with AE and metformin could significantly decrease the inflammatory factors (NF-κB p65, MCP-1, ICAM-1, IL-6) (Fig. 3i). The results revealed that metformin could reverse inflammatory factors to the levels of NC group, and moderate-dose AE (HF-OB group) was more effective to attenuate inflammation than high-dose AE (HF-OA group). As shown in Fig. 3, the phosphorylation of NF-κB p65 was markedly elevated in aortas of HFD group, while AE and metformin could reverse it and metformin was more efficient (Fig. 3j). Furthermore, we treated HAECs with PA for 19 h to investigate the effects of AE and metformin on inflammation in vitro. We compared the effects of different concentrations of AE and metformin on inflammatory response in HAECs. The 150 μg/ml AE and 2 mmol/l metformin could reverse the inflammatory factors most effectively without influence on the status of HAECs, so we choose 150 μg/ml AE and 2 mM metformin in the following experiments (Supplemental Figure 3). Consistent with the results in rats, the inflammatory factors and the phosphorylation of NF-κB p65 were significantly up-regulated by PA compared with NC group. In comparison, AE and metformin obviously inhibited the PA-induced inflammatory response in HAECs (Fig. 3k, l).

Therefore, AE and metformin could prevent high fat-induced fat accumulation, dyslipidemia and endothelial inflammation.

### AE and metformin regulate miR-146a and miR-155 as well as their target genes in high fat treated endothelium

**Fig. 4:**
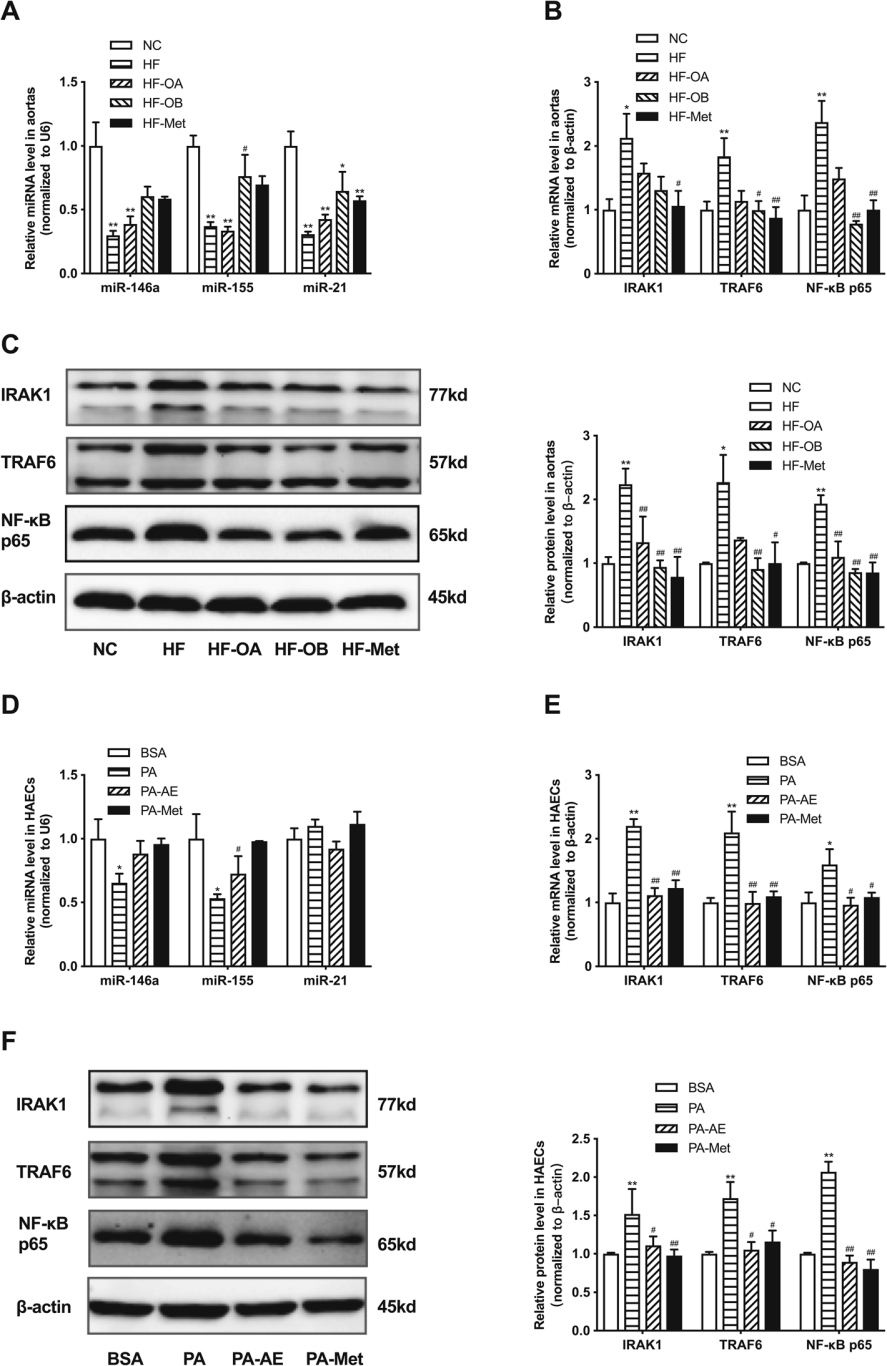
AE and metformin regulate miR-146a and miR-155 as well as their target genes in high fat treated aortas and HAECs. **a**, relative expression levels of miR-146a, miR-155 and miR-21 in rat aortas. **b**, relative mRNA levels of IRAK1, TRAF6 and NF-κB p65 in rat aortas. **c**, relative protein levels of IRAK1, TRAF6 and NF-κB p65 in rat aortas. **d**, relative expression levels of miR-146a, miR-155 and miR-21 in HAECs. **e**, relative mRNA levels of IRAK1, TRAF6 and NF-κB p65 in HAECs. **f**, relative protein levels of IRAK1, TRAF6 and NF-κB p65 in HAECs. Data are presented as mean ± SEM. ^*^P < 0.05 versus NC or BSA group. ^**^P < 0.01 versus NC or BSA group. ^#^*P*<0.05 versus HF or PA group. ^*#*#^*P*<0.01 versus HF or PA group. *n* = 6–8 rats per group for animal study. *n* = 5 per group for HAECs study

To determine whether the inhibitory effects of AE and metformin on inflammation is depended on the regulation of miRNAs, we examined the expressions of miRNAs related with inflammation both in vivo and in vitro. Compared with NC group, the relative expressions of miR-146a, miR-155, and miR-21 were decreased in aortas of HF group. Importantly, AE and metformin treatments resulted in increases in the expressions of miR-146a and miR-155 in HF-OB group and HF-Met group. However, neither AE nor metformin exhibited effects on the expression of miR-21 (Fig. 4a). Moreover, the mRNA and protein levels of the target genes (IRAK1,TRAF6 and NF-κB p65) were significantly decreased when rats treated with HFD were orally administrated with AE or metformin compared with HF group (Fig. 4b, c). Moreover, it showed that moderate-dose AE (HF-OB group) is more effective than high-dose AE (HF-OA group) in increasing the expressions of miR-146a and miR-155 as well as decreasing their target genes, which suggested that the concentration of AE may be involved in its effect on miRNAs. And there were no obvious differences between HF-OB group and HF-Met group.

Treatment with AE and metformin could reverse the expressions of miR-146a and miR-155 but not miR-21 to the normal level in PA-stimulated HAECs (Fig. 4d). Additionally, the mRNA and protein expressions of IRAK1, TRAF6 and NF-κB p65 were up-regulated when exposure HAECs to PA, which showed decreases after treated with AE or metformin (Fig. 4e, f). Thus, we assume that AE and metformin may improve the inflammatory response in endothelium via up-regulating miR-146a and miR-155 as well as inhibiting their target genes.

### The inflammation-inhibitory roles of AE and metformin are depended on miR-146a in endothelial cells

**Fig. 5:**
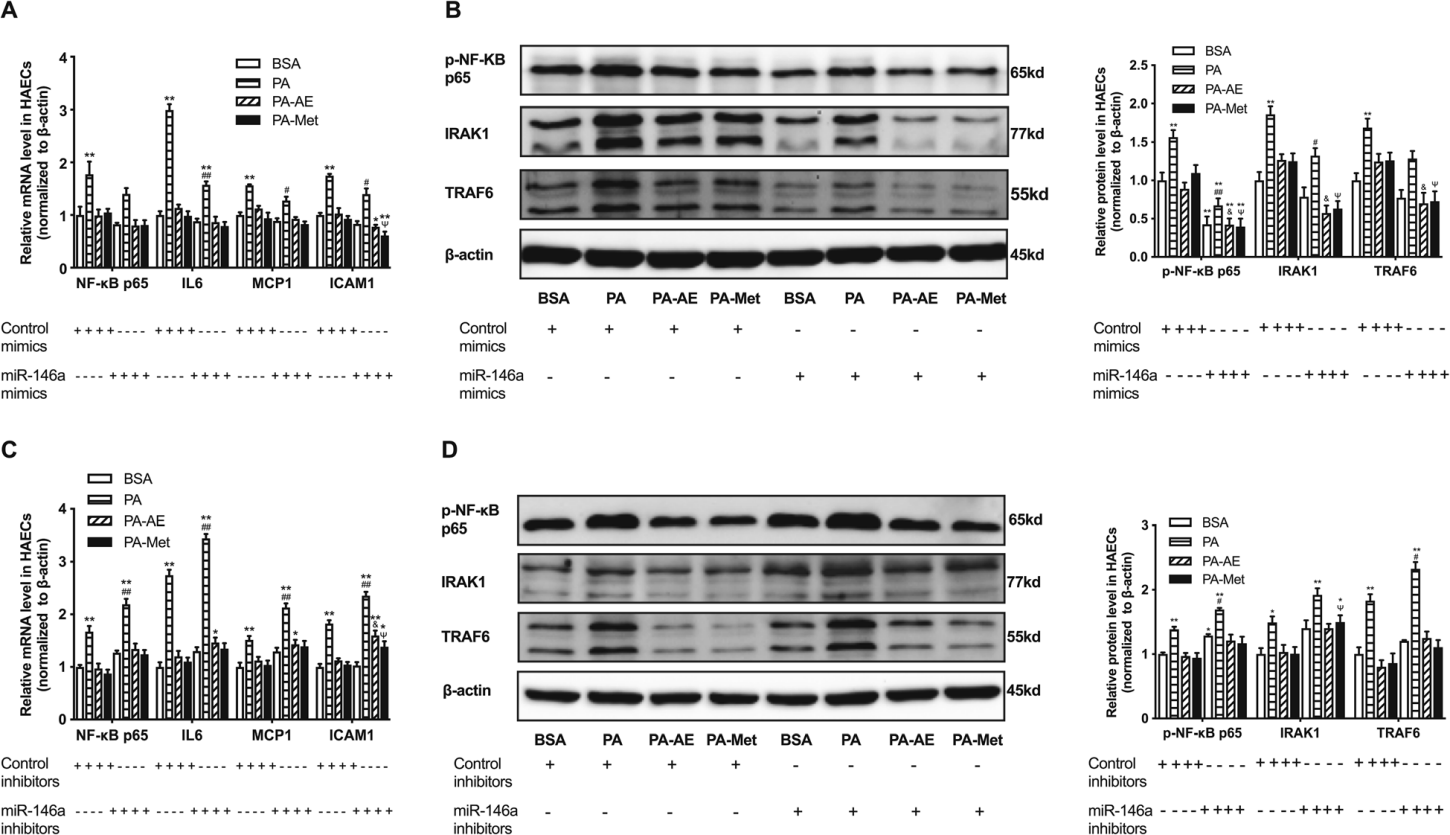
AE and metformin inhibit high fat induced endothelial inflammation via regulating miR-146a and its target genes in HAECs. **a**, relative mRNA levels of NF-κB p65, IL-6, MCP-1 and ICAM-1 in HAECs transfected with miR-146a mimics. **b**, relative protein levels of p-NF-κB p65, IRAK1 and TRAF6 in HAECs transfected with miR-146a mimics. **c**, relative mRNA levels of NF-κB p65, IL-6, MCP-1 and ICAM-1 in HAECs transfected with miR-146a inhibitors. **d**, relative protein levels of p-NF-κB p65, IRAK1 and TRAF6 in HAECs transfected with miR-146a inhibitors. Data are presented as mean ± SEM. ^*^P < 0.05 versus BSA group. ^**^P < 0.01 versus BSA group. ^#^*P*<0.05 versus control mimics or inhibitors in PA group. ^*#*#^*P*<0.01 versus control mimics or inhibitors in PA group. ^&^*P*<0.05 versus control mimics or inhibitors in PA-AE group. ^Ψ^*P*<0.05 versus control mimics or inhibitors in PA-Met group. *n* = 5 per group for HAECs study

miR-146a was found to be down-regulated by high fat, while AE and metformin could modulate its expression both in vivo and in vitro. To examine whether miR-146a mediates the protective role of AE and metformin, the miR-146a mimics and inhibitors were transfected into HAECs. As shown in Supplemental Figure 4A, miR-146a mimics resulted in a significant increase in the expression of miR-146a compared with control mimics. The protein levels of IRAK1 and TRAF6, as the target genes of miR-146a, were both repressed by miR-146a mimics compared with control mimics in HAECs (Fig. 5b). The expressions of inflammatory factors (NF-κB p65, IL-6, MCP-1 and ICAM-1) and the phosphorylation level of NF-κB p65 were reduced after miR-146a mimics transfection into HAECs (Fig. 5a, b). In addition, the inflammatory factors and target genes were further inhibited by miR-146a mimics in PA-AE and PA-Met groups compared with control mimics transfected group (Fig. 5a, b).

By comparison, the expression of miR-146a were effectively reduced in miR-146a inhibitors transfected group (Supplemental Figure 4B). The protein levels of IRAK1 and TRAF6 were elevated by miR-146a inhibitors when compared with control inhibitors (Fig. 5d). Besides, miR-146a inhibitors induced up-regulation of inflammatory factors (NF-κB p65, IL-6, MCP-1 and ICAM-1) and the phosphorylation level of NF-κB p65 compared with corresponding groups transfected with control inhibitors (Fig. 5c, d). Furthermore, inhibition of miR-146a deteriorated the endothelial inflammation in PA-treated group compared with control inhibitors. Meanwhile, AE and metformin mediated improved inflammation were weakened by miR-146a inhibitors transfection (Fig. 5c, d). These results suggest that miR-146a participate in high fat-induced inflammation in endothelium through directly regulating IRAK1 and TRAF6, and miR-146a may be indispensable for AE and metformin mediated the improvement of inflammation.

### The inflammation-inhibitory roles of AE and metformin are depended on miR-155 in endothelial cells

**Fig. 6:**
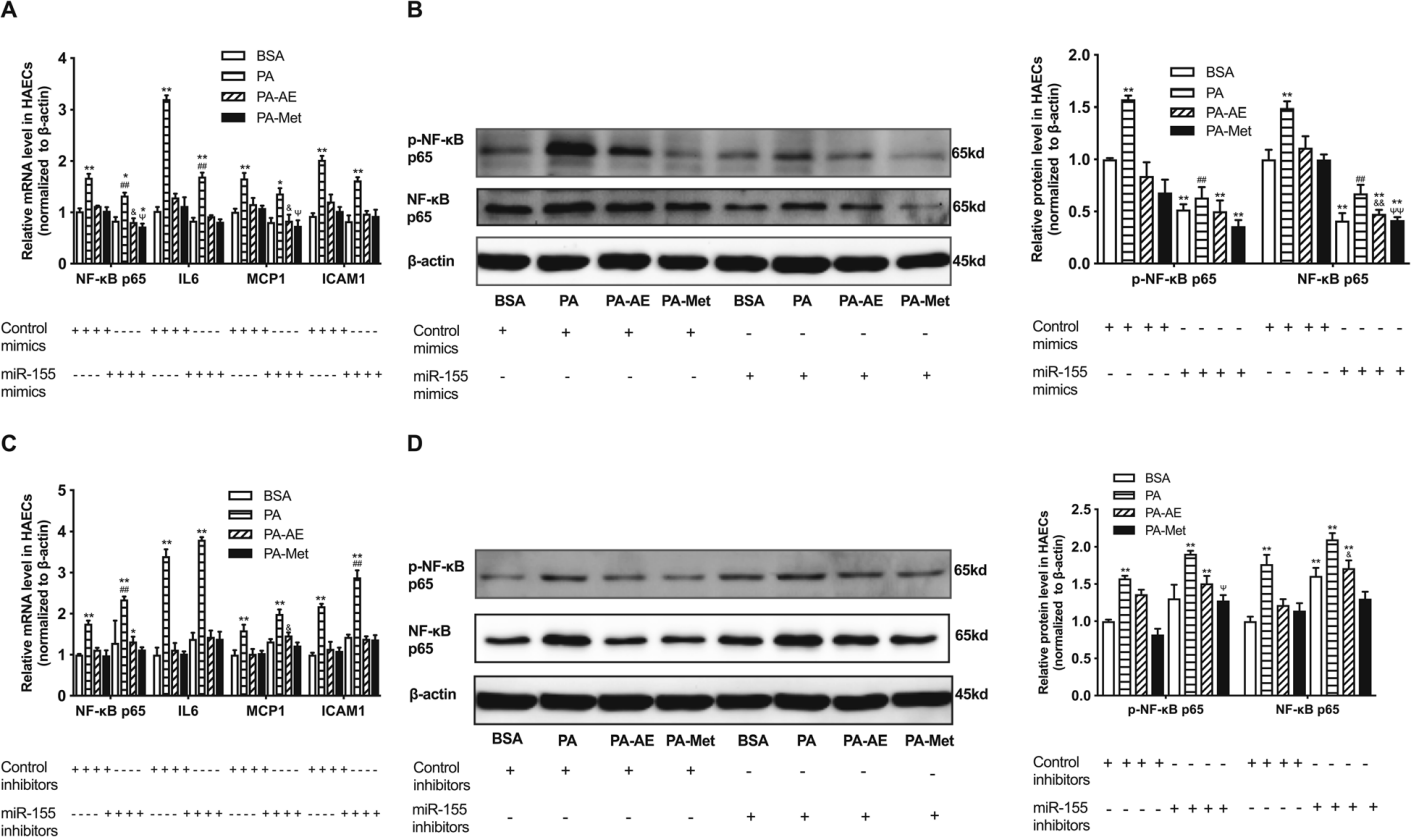
AE and metformin inhibit high fat-induced endothelial inflammation via regulating miR-155 and its target gene NF-κB p65 in HAECs. **a**, relative mRNA levels of NF-κB p65, IL-6, MCP-1 and ICAM-1 in HAECs transfected with miR-155 mimics. **b**, relative protein levels of p-NF-κB p65 and NF-κB p65 in HAECs transfected with miR-155 mimics. **c**, relative mRNA levels of NF-κB p65, IL-6, MCP-1 and ICAM-1 in HAECs transfected with miR-155 inhibitors. **d**, relative protein levels of p-NF-κB p65 and NF-κB p65 in HAECs transfected with miR-155 inhibitors. Data are presented as mean ± SEM. ^*^P < 0.05 versus BSA group. ^**^P < 0.01 versus BSA group. ^#^*P*<0.05 versus control mimics or inhibitors in PA group. ^*#*#^*P*<0.01 versus control mimics or inhibitors in PA group. ^&^*P*<0.05 versus control mimics or inhibitors in PA-AE group. ^&&^*P*<0.01 versus control mimics or inhibitors in PA-AE group. ^Ψ^*P*<0.05 versus control mimics or inhibitors in PA-Met group. ^ΨΨ^*P*<0.01 versus control mimics or inhibitors in PA-Met group. *n* = 5 per group for HAECs study

To further investigate whether the inhibitory effects of AE and metformin on high fat-induced inflammation in aortas and HAECs is depended on its regulation on miR-155, we transfected miR-155 mimics or inhibitors into HAECs (Supplemental Figure 4C and 4D). As the target gene of miR-155, the mRNA and protein levels of NF-κB p65 were down-regulated by miR-155 mimics (Fig. 6a, b). Meanwhile, the miR-155 mimics reduced the mRNA levels of inflammatory factors (IL-6, MCP-1 and ICAM-1) and the phosphorylation level of NF-κB p65 when compared with control mimics in HAECs (Fig. 6a, b). In addition, miR-155 mimics further suppressed the inflammatory factors and target genes in PA-AE and PA-Met group compared with control mimics (Fig. 6a, b).

In comparison, the mRNA and protein levels of NF-κB p65 were up-regulated by miR-155 inhibitors (Fig. 6c, d). The expressions of inflammatory factors (IL-6, MCP-1 and ICAM-1) and the phosphorylation level of NF-κB p65 were increased by miR-155 inhibitors compared with control inhibitors (Fig. 6c, d). Additionally, the elevated inflammatory factors and target genes induced by PA were exacerbated by miR-155 inhibitors in PA group, and miR-155 inhibitors abolished the benefit effects of AE and metformin on inflammation in PA-AE and PA-Met group (Figs. 5d and 6c). Taken together, these results indicate that miR-155 plays an important role in inflammation activation induced by high fat via the direct regulation on NF-κB p65, AE and metformin could protect against high fat-induced inflammation though regulation of miR-155 in endothelium.

### The inflammation-inhibitory roles of AE and metformin is independent of miR-21 in endothelial cells

**Fig. 7:**
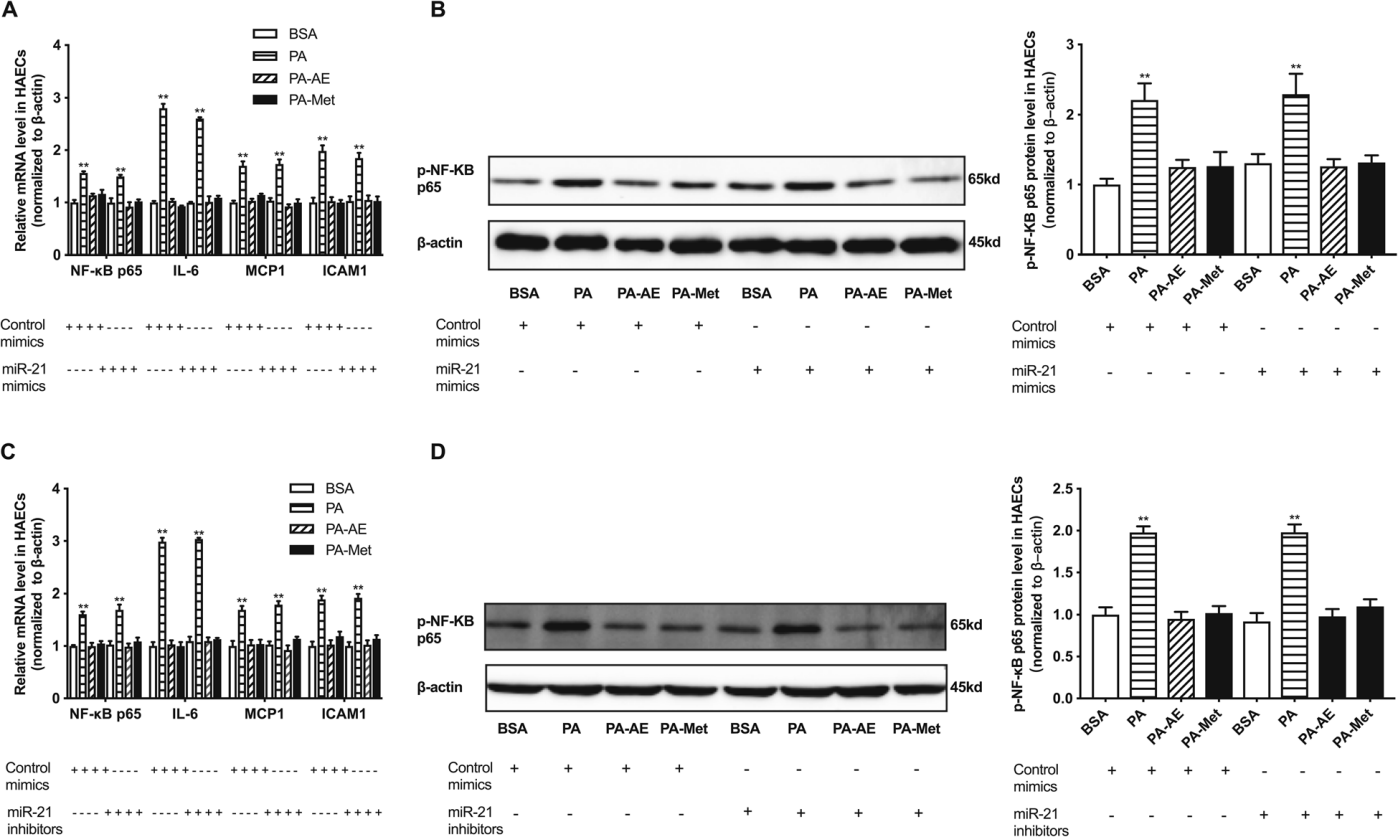
AE and metformin inhibit high fat-induced endothelial inflammation independent of miR-21 in PA-exposed HAECs. **a**, relative mRNA levels of NF-κB p65, IL-6, MCP-1 and ICAM-1 in HAECs transfected with miR-21 mimics. **b**, relative protein level of p-NF-κB p65 in HAECs transfected with miR-21 mimics. **c**, relative mRNA levels of NF-κB p65, IL-6, MCP-1 and ICAM-1 in HAECs transfected with miR-21 inhibitors. **d**, relative protein levels of p-NF-κB p65 in HAECs transfected with miR-21 inhibitors. Data are presented as mean ± SEM. ^*^P < 0.05 versus BSA group. ^**^P < 0.01 versus BSA group. *n* = 5 per group for HAECs study

In this study, the expression of miR-21 was decreased in aortas from HF group, while it showed no difference in PA-exposed HAECs. In addition, neither AE nor metformin exhibited effect on the expression of miR-21 in aortas of HF rats and PA-stimulated HAECs, indicating that miR-21 may not be involved in high fat-induced endothelial inflammation. To validate this hypothesis, we transfected miR-21 mimics and inhibitors into HAECs (Supplemental Figure 4E, 4F). The results showed that inflammatory factors were activated in PA group as well as the phosphorylation of NF-κB p65. However, neither miR-21 mimics nor inhibitors exhibited effect on inflammatory factors and the phosphorylation level of NF-κB p65 compared with control mimics or inhibitors in HAECs (Fig. 7). In brief, the results suggest that miR-21 may not participate in high fat-induced inflammation in HAECs. AE and metformin mediated inflammatory improvement is not dependent on miR-21.

## Discussion

This study found that decreased miR-146a and miR-155 contribute to vascular endothelial inflammation induced by high fat. In addition, AE and metformin could attenuate the endothelial inflammation via up-regulating miR-146a and miR-155. However, miR-21 are not involved in high-fat induced vascular endothelial inflammation.

Obesity increases the risk of cardiovascular morbidity and mortality. It is well-known that obesity is associated with low-grade inflammation [25]. Inflammation plays a key role in the development of vascular diseases, such as atherosclerosis, myocardial infarction, and stroke. Endothelial inflammation plays a critical role in the initiation and progression of atherosclerosis [26]. The Canakinumab Antiinflammatory Thrombosis Outcome Study (CANTOS study) demonstrated that anti-inflammatory therapy with canakinumab, which targets IL-1β innate immunity pathway, resulted in a significant decreasing risk of recurrent cardiovascular events. What’s more, the beneficial effect was independent of lipid lowering [27]. This study directly proved the inflammatory hypothesis in atherosclerosis. In our study, the inflammatory factors (NF-κB p65, MCP-1, ICAM-1 and IL-6) as well as the phosphorylation of NF-κB p65 were all activated in high fat-treated aortas and endothelial cells.

As the important part of epigenetics, miRNAs have been identified as critical regulator of endothelial inflammation [28, 29]. miR-146a emerged as negative regulators of the immune response [30]. However, the role of miR-146a is poorly understood in endothelium treated with high fat. As inflammation suppressor, miR-146a has been reported in many diseases, such as diabetes, rheumatoid arthritis, chronic renal inflammation, atopic dermatitis, and postpartum psychosis [31–35]. It is reported that apoE increased expression of miR-146a to suppress NF-κB-driven inflammation and atherosclerosis in macrophages and monocytes [36], which implies that miR-146a suppresses inflammation during hyperlipidemia to attenuate atherosclerosis. Additionally, miR-146a was found to repress inflammation in endothelial cells upon exposure to pro-inflammatory cytokines [5]. Previously, we demonstrated that miR-146a was involved in NF-κB pathway activation in condition of hyperglycemia. MiR-146a could bind to the 3’UTR of IRAK1 and TRAF6 to suppress their expressions by luciferase reporter assay [9]. Consistently, it showed that high fat decreased the expression of miR-146a in aortas and HAECs, as well as activating inflammation response in this study. High fat could still increase the target genes of miR-146a, IRAK1 and TRAF6. Moreover, miR-146a mimics transfection could repress the inflammation activation by targeting IRAK1 and TRAF6, while miR-146a inhibitors exhibited the opposite effect. The results reveal that miR-146a is involved in inflammation activation through directly regulating IRAK1 and TRAF6 in high fat treated endothelium.

miR-155, as a typical multi-functional miRNA, plays conflicting roles in the pathogenesis of cardiovascular diseases [37]. Results of studies investigating the effect of miR-155 on inflammation in monocytes or macrophages displayed a conflicting role of miR-155. miR-155 silencing in human THP-1 macrophages significantly enhanced oxLDL-induced lipid uptake and inflammation response via targeting NF-κB [38], indicating that miR-155 can negatively regulate inflammation. However, it is noted that loss of miR-155 reduced the expression of chemokine in macrophages stimulated with oxLDL/IFN-γ, suggesting that miR-155 exhibits pro-inflammatory effect in macrophages via suppressing B cell leukemia/lymphoma 6 (BCL6) [7]. Circulating miR-155 level was reported to decrease in patients with coronary artery disease compared with healthy controls [39]. Additionally, it is reported that miR-155 could inhibit inflammation and migration in human umbilical vein endothelial cells (HUVECs) when exposure to angiotensin-II by targeting AT1R [40]. These studies imply the anti-inflammation effect of miR-155 in cardiovascular diseases. So far, several direct targets of miR-155 have been identified by luciferase report assay, such as suppressors of cytokine signaling 1 (SOCS1), src homology 2 domain-containing inositol phosphatase 1 (SHIP1), interleukin 13 receptor alpha 1 (IL13Rα1) and SMAD family member 2 (SMAD2) in macrophages as well as BCL6, which acts as NF-κB antagonist [41]. In endothelial cells, NF-κB p65 was investigated as the direct target of miR-155 by luciferase report assay [10]. In our data, miR-155 was reduced by high fat stimulation in endothelium along with activation of inflammation response. When transfection with miR-155 mimics, the inflammation was alleviated in HAECs in presence of high fat. Whereas, miR-155 inhibitors exacerbated the inflammation response induced by high fat in HAECs. The contradictory roles of miR-155 in cardiovascular diseases may be on account of different pathological stages or animal models of the diseases. Our results suggested that miR-155 may play an important role in inhibiting endothelial inflammation response through suppression of NF-κB p65 during high fat diet.

miR-21 is identified to be involved in cardiovascular diseases and play a key role in regulating inflammation [42]. But whether miR-21 plays pro or anti-inflammation role still remains conflicting. By using microarray, the expression of miR-21 was found to be elevated in human advanced coronary atherosclerotic plaques and serum from acute coronary syndrome patients, respectively [43, 44]. These results indicate the important role of miR-21 in cardiovascular diseases. It is reported that miR-21 was up-regulated by oscillatory shear stress, and promoted AP-1 activation, the expressions of pro-inflammatory cytokine VCAM-1 and MCP-1, as well as the monocytes adhesion to endothelial cells [45]. This indicated the pro-inflammatory role of miR-21 in endothelial cells, thus accelerating the process of atherosclerosis. However, another study found that miR-21 deficiency in macrophage promoted apoptosis, plaque necrosis and vascular inflammation [6]. Additionally, miR-21 was reported to suppress inflammation and cellular apoptosis, thus attenuating leakage of injured microvascular endothelial barrier in brain [46]. These reports provide the evidence that miR-21 could inhibit vascular inflammation and improve atherogenesis. In our data, miR-21 was decreased in rat aortas treated with high fat compared with normal control, while its expression was not changed in PA treated HAECs. There is a complex process in vivo, the decreased expression of miR-21 in vivo may be due to other factors or as an indirect response in high fat treatment. So, it suggests that miR-21 may not participate in high fat induced endothelial inflammation.

Growing evidences have shown that metformin could reduce endothelial inflammation [21, 22, 47, 48]. The extract of AE could attenuate vascular impairment and reduce the levels of inflammatory factors in load-induced fatigued rats [18, 49]. Here we suppose that AE and Metformin may improve high fat induced endothelial inflammation by regulating miR-146a and miR-155. AE is rich in flavonoid and polysaccharides compounds. Epidemiological studies have revealed that consumption of food rich in flavonoid compounds displays beneficial effect on the risk of diabetes, obesity, hyperlipidemia, cardiovascular diseases and cancer [50–52]. In animal studies, it is reported AE/its extracts could decrease blood glucose and lipid level in high fat diet induced obese animals as well as in diabetic animals [11, 12, 14–16, 53]. Furthermore, AE was reported to improve islet structure in diabetes by PPAR-dependent mechanism [54], and active subfractions of AE substantially attenuated free fatty acid-induced β cell apoptosis through inhibiting dipeptidyl peptidase-4 [55]. Despite glucose and lipid metabolism, Huangkui and lectin, the extracts of AE, were found to inhibit inflammation response in kidney and temporomandibular joint [49, 56]. Additionally, quercetin-3-O-gentiobiose, the extract of AE, had anti-fatigue and vasoprotective effects through enhancing the activities of antioxidant enzymes and improving the levels of inflammatory cytokines, thus attenuating vascular endothelial dysfunction induced by endurance swimming [18]. Despite the multiple protective functions of AE on metabolism and vascular system, the specific mechanism of AE in vasoprotection still remains unclear. In our study, AE could improve the systematic metabolic disorders induced by high fat, such as body weight gain, impaired fasting glucose and abnormal lipid profile. This may be contributed to the flavonoid compounds. And the protective role in vascular inflammation may be owing to huangkui, lectin and quercetin-3-O-gentiobiose, because they could alleviate the inflammatory response in various diseases. However, the specific compounds responsible for the protective effect in metabolism and vascular inflammation still need further investigation. In agreement with previous studies, our results showed that AE inhibited inflammatory factors as wells as the phosphorylation level of NF-κB p65 in high fat treated aortas of rats and HAECs through up-regulation of miR-146a and miR-155. What’s more, the moderate-dose AE (400 mg/kg) was more effective than high-dose AE (800 mg/kg), suggesting the inhibitory effect on endothelial inflammation of AE is dose-dependent. Therefore, AE may protect endothelial function by regulation of miR-146a and miR-155 as well as their targets.

Metformin is the first-line drug in type 2 diabetes mainly by improving insulin sensitivity. In addition to reducing hyperglycemia and enhancing insulin sensitivity, it has been suggested that metformin improves endothelial function and promotes vasoprotection [57]. The United Kingdom Prospective Diabetes Study (UKPDS) clinical trial showed that metformin treatment decreases the risk of cardiovascular diseases endpoints in overweight newly diagnosed with type 2 diabetes [58]. Moreover, metformin has been reported to restore endothelial function in diabetic status [22, 24, 47]. In addition, there are evidences supporting an anti-inflammatory effect of metformin particularly in obesity, diabetes and atherosclerosis [21, 48, 59, 60]. Similarly, our results suggested that metformin suppressed inflammatory response induced by high-fat in endothelium. However, other studies revealed that treatment with metformin had no effect on inflammation in diabetes or impaired glucose tolerance patients [24, 47]. It is uncertain whether the beneficial effects of metformin are due to a direct effect or the results of improved insulin sensitivity, weight loss and improved hyperglycemia. The specific mechanism of metformin on vascular system protection has not been fully elucidated. We showed that metformin could increase the expressions of miR-146a and miR-155 as well as regulation of their targets (IRAK1, TRAF6 and NF-κB p65) to improve inflammation responses in endothelium.

## Conclusions

Our study demonstrates that miR-146a and miR-155 are involved in the high fat-induced inflammation in endothelium. The evidence suggests that miRNAs could explain the endothelial inflammation in obesity. Additionally, AE and metformin improve the inflammation response via up-regulations of miR-146a and miR-155 in endothelium. Moreover, miR-146a and miR-155 may participate the pathogenesis of cardiovascular diseases induced by obesity. The regulation of miR-146a and miR-155 is one of the mechanisms underlying the vasoprotective effects of AE and metformin.

## Supplementary information

**Additional file 1 **: **Figure S1.** HFD induced increases in body weight, fat deposition, impaired fasting blood glucose and dyslipidemia. **Figure S2.** Regulating miR-21 have no effects on high fat induced endothelial inflammation in HAECs. **Figure S3.** The effects of different concentrations of AE and metformin on the inflammation factors in HAECs after treatment with PA. **Figure S4.** The relative expressions of miRNAs in HAECs after transfection with mimics or inhibitors. **Table S1.** Primers for RT-PCR. **Table S2.** The primary antibodies for Western Blotting.

## Data Availability

The datasets used and/or analysed during the current study are available from the corresponding author on reasonable request.

## Abbreviations

miRNAs: microRNAs
IRAK1: IL-1 receptor-associated kinase 1
TRAF6: TNF receptor-associated factor 6
NF-κB p65: Nuclear factor-κB p65
AE: *Abelmoschus esculentus*
HAECs: Human aortic endothelial cells
HFD: High fat diet
SAT: Subcutaneous adipose tissue
VAT: Visceral adipose tissue
TC: Total cholesterol
TG: Triacylglycerol
LDL-C: Low density lipoprotein cholesterol
HDL-C: High density lipoprotein cholesterol
PA: Palmitic acid
MCP-1: Monocyte chemotactic protein 1
ICAM-1: intercellular cell adhesion molecule-1
IL-6: Interleukin 6

## References

[1] Wang Y, Qian Y, Fang Q (2017). Saturated palmitic acid induces myocardial inflammatory injuries through direct binding to TLR4 accessory protein MD2. Nat Commun.

[2] Sena CM, Pereira A, Fernandes R (2017). Adiponectin improves endothelial function in mesenteric arteries of rats fed a high-fat diet: role of perivascular adipose tissue. Br J Pharmacol.

[3] Gimbrone MA, Garcia-Cardena G (2013). Vascular endothelium, hemodynamics, and the pathobiology of atherosclerosis. Cardiovasc Pathol.

[4] Rebane A, Akdis CA (2013). MicroRNAs: essential players in the regulation of inflammation. J Allergy Clin Immunol.

[5] Cheng HS, Sivachandran N, Lau A (2013). MicroRNA-146 represses endothelial activation by inhibiting pro-inflammatory pathways. EMBO Mol Med.

[6] Canfran-Duque A, Rotllan N, Zhang X (2017). Macrophage deficiency of miR-21 promotes apoptosis, plaque necrosis, and vascular inflammation during atherogenesis. EMBO Mol Med.

[7] Nazari-Jahantigh M, Wei Y, Noels H (2012). MicroRNA-155 promotes atherosclerosis by repressing Bcl6 in macrophages. J Clin Invest.

[8] Gao M, Wang X, Zhang X (2015). Attenuation of cardiac dysfunction in Polymicrobial Sepsis by MicroRNA-146a is mediated via targeting of IRAK1 and TRAF6 expression. J Immunol.

[9] Zhong X, Liao Y, Chen L (2015). The MicroRNAs in the pathogenesis of metabolic memory. Endocrinology.

[10] Wu XY, Fan WD, Fang R (2014). Regulation of microRNA-155 in endothelial inflammation by targeting nuclear factor (NF)-kappaB P65. J Cell Biochem.

[11] Mishra N, Kumar D, Rizvi SI (2016). Protective effect of Abelmoschus esculentus against Alloxan-induced diabetes in Wistar strain rats. J Diet Suppl.

[12] Khosrozadeh M, Heydari N, Abootalebi M (2016). The effect of Abelmoschus Esculentus on blood levels of glucose in diabetes mellitus. Iran J Med Sci.

[13] Peng CH, Chyau CC, Wang CJ (2016). Abelmoschus esculentus fractions potently inhibited the pathogenic targets associated with diabetic renal epithelial to mesenchymal transition. Food Funct.

[14] Fan S, Guo L, Zhang Y (2013). Okra polysaccharide improves metabolic disorders in high-fat diet-induced obese C57BL/6 mice. Mol Nutr Food Res.

[15] Sabitha V, Ramachandran S, Naveen KR (2011). Antidiabetic and antihyperlipidemic potential of Abelmoschus esculentus (L.) Moench. In streptozotocin-induced diabetic rats. J Pharm Bioallied Sci.

[16] Fan S, Zhang Y, Sun Q (2014). Extract of okra lowers blood glucose and serum lipids in high-fat diet-induced obese C57BL/6 mice. J Nutr Biochem.

[17] Tian ZH, Miao FT, Zhang X (2015). Therapeutic effect of okra extract on gestational diabetes mellitus rats induced by streptozotocin. Asian Pac J Trop Med.

[18] Lin Y, Liu HL, Fang J (2014). Anti-fatigue and vasoprotective effects of quercetin-3-O-gentiobiose on oxidative stress and vascular endothelial dysfunction induced by endurance swimming in rats. Food Chem Toxicol.

[19] Cameron AR, Morrison VL, Levin D (2016). Anti-inflammatory effects of metformin irrespective of diabetes status. Circ Res.

[20] Calixto MC, Lintomen L, Andre DM (2013). Metformin attenuates the exacerbation of the allergic eosinophilic inflammation in high fat-diet-induced obesity in mice. PLoS One.

[21] Xu W, Deng YY, Yang L (2015). Metformin ameliorates the proinflammatory state in patients with carotid artery atherosclerosis through sirtuin 1 induction. Transl Res.

[22] Sena CM, Matafome P, Louro T (2011). Metformin restores endothelial function in aorta of diabetic rats. Br J Pharmacol.

[23] Hattori Y, Suzuki K, Hattori S (2006). Metformin inhibits cytokine-induced nuclear factor kappaB activation via AMP-activated protein kinase activation in vascular endothelial cells. Hypertension.

[24] Caballero AE, Delgado A, Aguilar-Salinas CA (2004). The differential effects of metformin on markers of endothelial activation and inflammation in subjects with impaired glucose tolerance: a placebo-controlled, randomized clinical trial. J Clin Endocrinol Metab.

[25] Van Gaal LF, Mertens IL, De Block CE (2006). Mechanisms linking obesity with cardiovascular disease. Nature.

[26] Moore KJ, Tabas I (2011). Macrophages in the pathogenesis of atherosclerosis. Cell.

[27] Ridker PM, Everett BM, Thuren T (2017). Antiinflammatory therapy with Canakinumab for atherosclerotic disease. N Engl J Med.

[28] Hulsmans M, De Keyzer D, Holvoet P (2011). MicroRNAs regulating oxidative stress and inflammation in relation to obesity and atherosclerosis. FASEB J.

[29] Hulsmans M, Holvoet P (2013). MicroRNA-containing microvesicles regulating inflammation in association with atherosclerotic disease. Cardiovasc Res.

[30] Testa U, Pelosi E, Castelli G (2017). miR-146 and miR-155: two key modulators of immune response and tumor development. Noncoding. RNA.

[31] Ichii O, Otsuka S, Sasaki N (2012). Altered expression of microRNA miR-146a correlates with the development of chronic renal inflammation. Kidney Int.

[32] Zhou Q, Haupt S, Kreuzer JT (2015). Decreased expression of miR-146a and miR-155 contributes to an abnormal Treg phenotype in patients with rheumatoid arthritis. Ann Rheum Dis.

[33] Weigelt K, Bergink V, Burgerhout KM (2013). Down-regulation of inflammation-protective microRNAs 146a and 212 in monocytes of patients with postpartum psychosis. Brain Behav Immun.

[34] Rebane A, Runnel T, Aab A (2014). MicroRNA-146a alleviates chronic skin inflammation in atopic dermatitis through suppression of innate immune responses in keratinocytes. J Allergy Clin Immunol.

[35] Baldeon RL, Weigelt K, de Wit H (2014). Decreased serum level of miR-146a as sign of chronic inflammation in type 2 diabetic patients. PLoS One.

[36] Li K, Ching D, Luk FS (2015). Apolipoprotein E enhances microRNA-146a in monocytes and macrophages to suppress nuclear factor-kappaB-driven inflammation and atherosclerosis. Circ Res.

[37] Ma X, Ma C, Zheng X (2013). MicroRNA-155 in the pathogenesis of atherosclerosis: a conflicting role?. Heart Lung Circ.

[38] Huang RS, Hu GQ, Lin B (2010). MicroRNA-155 silencing enhances inflammatory response and lipid uptake in oxidized low-density lipoprotein-stimulated human THP-1 macrophages. J Investig Med.

[39] Fichtlscherer S, De Rosa S, Fox H (2010). Circulating microRNAs in patients with coronary artery disease. Circ Res.

[40] Zhu N, Zhang D, Chen S (2011). Endothelial enriched microRNAs regulate angiotensin II-induced endothelial inflammation and migration. Atherosclerosis.

[41] Wei Y, Nazari-Jahantigh M, Neth P (2013). MicroRNA-126, −145, and −155: a therapeutic triad in atherosclerosis?. Arterioscler Thromb Vasc Biol.

[42] Sheedy FJ (2015). Turning 21: induction of miR-21 as a key switch in the inflammatory response. Front Immunol.

[43] Parahuleva MS, Lipps C, Parviz B (2018). MicroRNA expression profile of human advanced coronary atherosclerotic plaques. Sci Rep.

[44] Darabi F, Aghaei M, Movahedian A (2017). Association of serum microRNA-21 levels with Visfatin, inflammation, and acute coronary syndromes. Heart Vessel.

[45] Zhou J, Wang KC, Wu W (2011). MicroRNA-21 targets peroxisome proliferators-activated receptor-alpha in an autoregulatory loop to modulate flow-induced endothelial inflammation. Proc Natl Acad Sci U S A.

[46] Ge X, Huang S, Gao H (2016). miR-21-5p alleviates leakage of injured brain microvascular endothelial barrier in vitro through suppressing inflammation and apoptosis. Brain Res.

[47] De Jager J, Kooy A, Lehert P (2005). Effects of short-term treatment with metformin on markers of endothelial function and inflammatory activity in type 2 diabetes mellitus: a randomized, placebo-controlled trial. J Intern Med.

[48] Jing Y, Wu F, Li D (2017). Metformin improves obesity-associated inflammation by altering macrophages polarization. Mol Cell Endocrinol.

[49] Freitas RS, do Val DR, Fernandes ME (2016). Lectin from Abelmoschus esculentus reduces zymosan-induced temporomandibular joint inflammatory hypernociception in rats via heme oxygenase-1 pathway integrity and tnf-alpha and il-1beta suppression. Int Immunopharmacol.

[50] Xiao ZP, Peng ZY, Peng MJ (2011). Flavonoids health benefits and their molecular mechanism. Mini-Rev Med Chem.

[51] Wedick NM, Pan A, Cassidy A (2012). Dietary flavonoid intakes and risk of type 2 diabetes in US men and women. Am J Clin Nutr.

[52] van Dam RM, Naidoo N, Landberg R (2013). Dietary flavonoids and the development of type 2 diabetes and cardiovascular diseases: review of recent findings. Curr Opin Lipidol.

[53] Huang CN, Wang CJ, Lin CL (2017). The nutraceutical benefits of subfractions of Abelmoschus esculentus in treating type 2 diabetes mellitus. PLoS One.

[54] Erfani Majd N, Tabandeh MR, Shahriari A (2018). Okra (Abelmoscus esculentus) improved islets structure, and down-regulated PPARs gene expression in pancreas of high-fat diet and Streptozotocin-induced diabetic rats. Cell J.

[55] Huang CN, Wang CJ, Lee YJ (2017). Active subfractions of Abelmoschus esculentus substantially prevent free fatty acid-induced beta cell apoptosis via inhibiting dipeptidyl peptidase-4. PLoS One.

[56] Tu Y, Sun W, Wan YG (2013). Huangkui capsule, an extract from Abelmoschus manihot (L.) medic, ameliorates adriamycin-induced renal inflammation and glomerular injury via inhibiting p38MAPK signaling pathway activity in rats. J Ethnopharmacol.

[57] Nesti L, Natali A (2017). Metformin effects on the heart and the cardiovascular system: a review of experimental and clinical data. Nutr Metab Cardiovasc Dis.

[58] Holman RR, Paul SK, Bethel MA (2008). 10-year follow-up of intensive glucose control in type 2 diabetes. N Engl J Med.

[59] Yang Q, Yuan H, Chen M (2018). Metformin ameliorates the progression of atherosclerosis via suppressing macrophage infiltration and inflammatory responses in rabbits. Life Sci.

[60] Fidan E, Onder Ersoz H, Yilmaz M (2011). The effects of rosiglitazone and metformin on inflammation and endothelial dysfunction in patients with type 2 diabetes mellitus. Acta Diabetol.

