# Supplementary material for: High fat-induced inflammation in vascular endothelium can be improved by Abelmoschus esculentus and metformin via increasing the expressions of miR-146a and miR-155
Source: Nutr Metab (Lond). 2020 May 13;17:35. doi: 10.1186/s12986-020-00459-7 (PMC7222555; doi:10.1186/s12986-020-00459-7)
Supplement: Supplementary file 1 — Additional file 1 : Figure S1. HFD induced increases in body weight, fat deposition, impaired fasting blood glucose and dyslipidemia. Figure S2. Regulating miR-21 have no effects on high fat induced endothelial inflammation in HAECs. Figure S3. The effects of different concentrations of AE and metformin on the inflammation factors in HAECs after treatment with PA. Figure S4. The relative expressions of miRNAs in HAECs after transfection with mimics or inhibitors. Table S1. Primers for RT-PCR. Table S2. The primary antibodies for Western Blotting. [file 12986_2020_459_MOESM1_ESM.docx]

**Supplemental Figure**

**
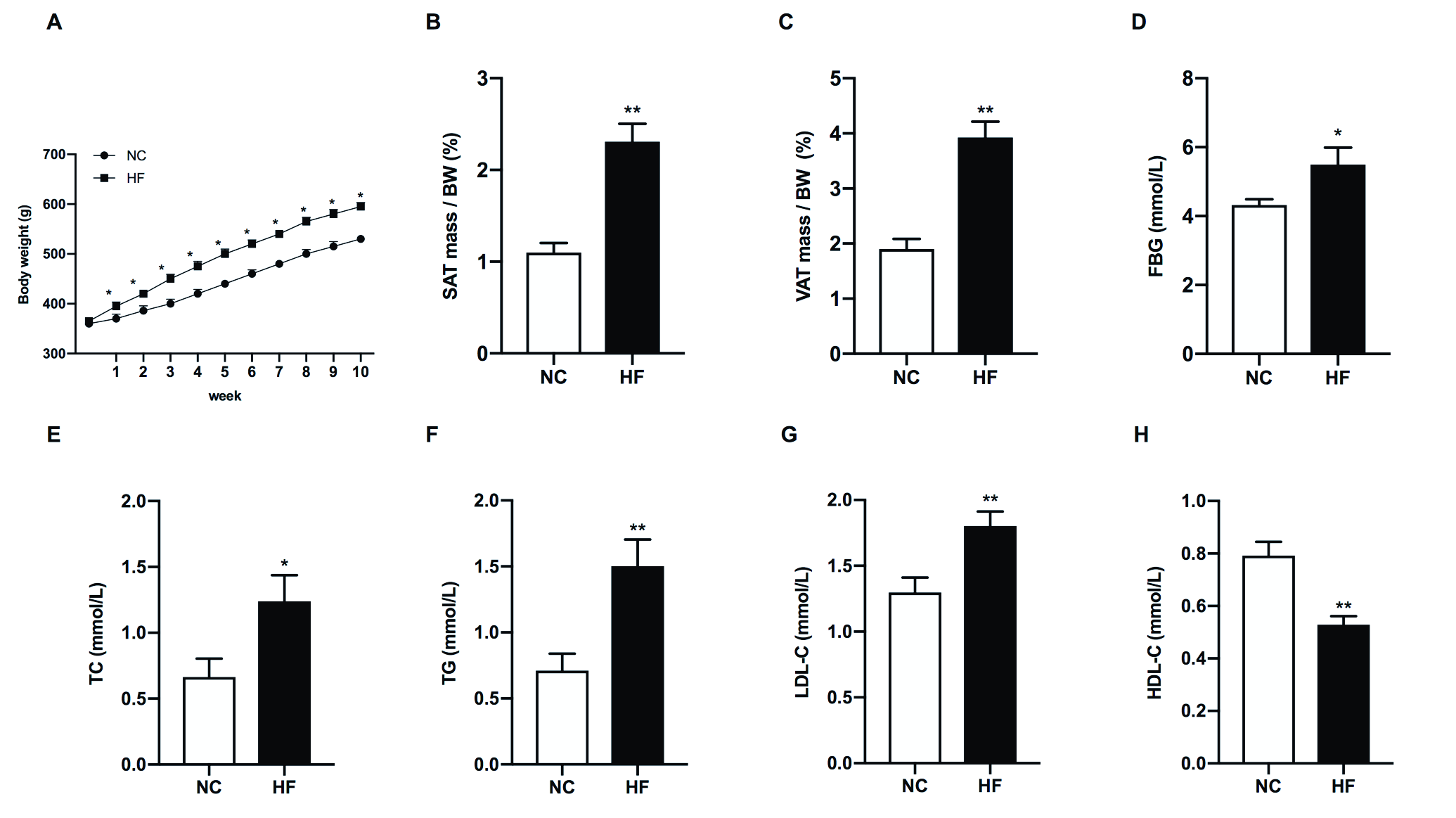
**

**Supplemental Figure 1.** **HFD induced increases in body weight, fat deposition, impaired fasting blood glucose and dyslipidemia.** A, body weight of rats after challenged with high fat diet for 10 weeks. B, the percent of subcutaneous fat in rats. C, the percent of visceral fat in rats. D, the fasting glucose level in rats. E-I, the TC, TG, LDL-C and HDL-C levels in serum of rats. Data are presented as mean±SEM. ^*^*P* < 0.05 versus NC group. ^**^*P* < 0.01 versus NC group. n=6-15 rats per group.


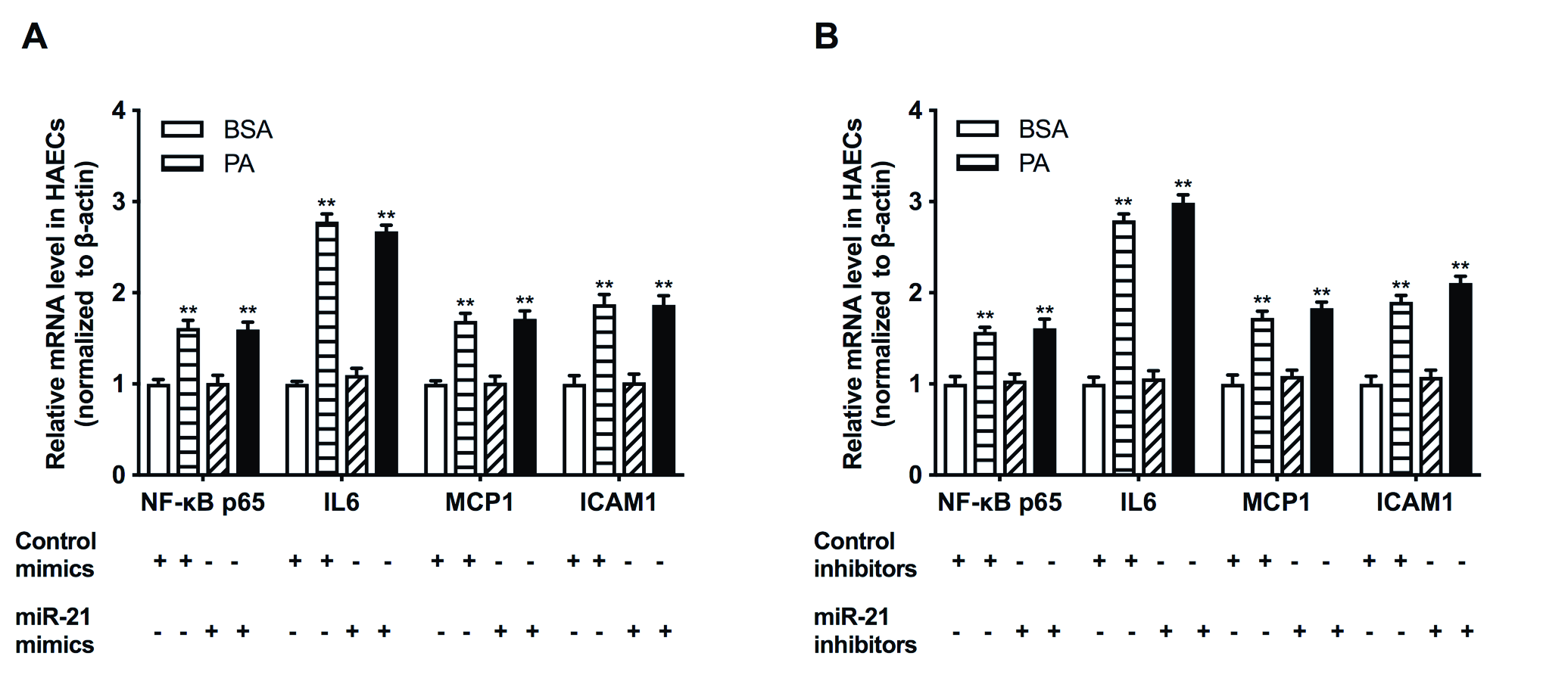


**Supplemental Figure 2.** **Regulating miR-21 have no effects on high fat induced endothelial inflammation in HAECs.** A-B, relative expression levels of inflammatory factors (NF-κB p65, IL-6, MCP-1 and ICAM-1) after transfection with miR-21 mimics and inhibitors, respectively. Data are presented as mean±SEM. ^*^*P* < 0.05 versus BSA group. ^**^*P* < 0.01 versus BSA group. n=5 per group for HAECs study.


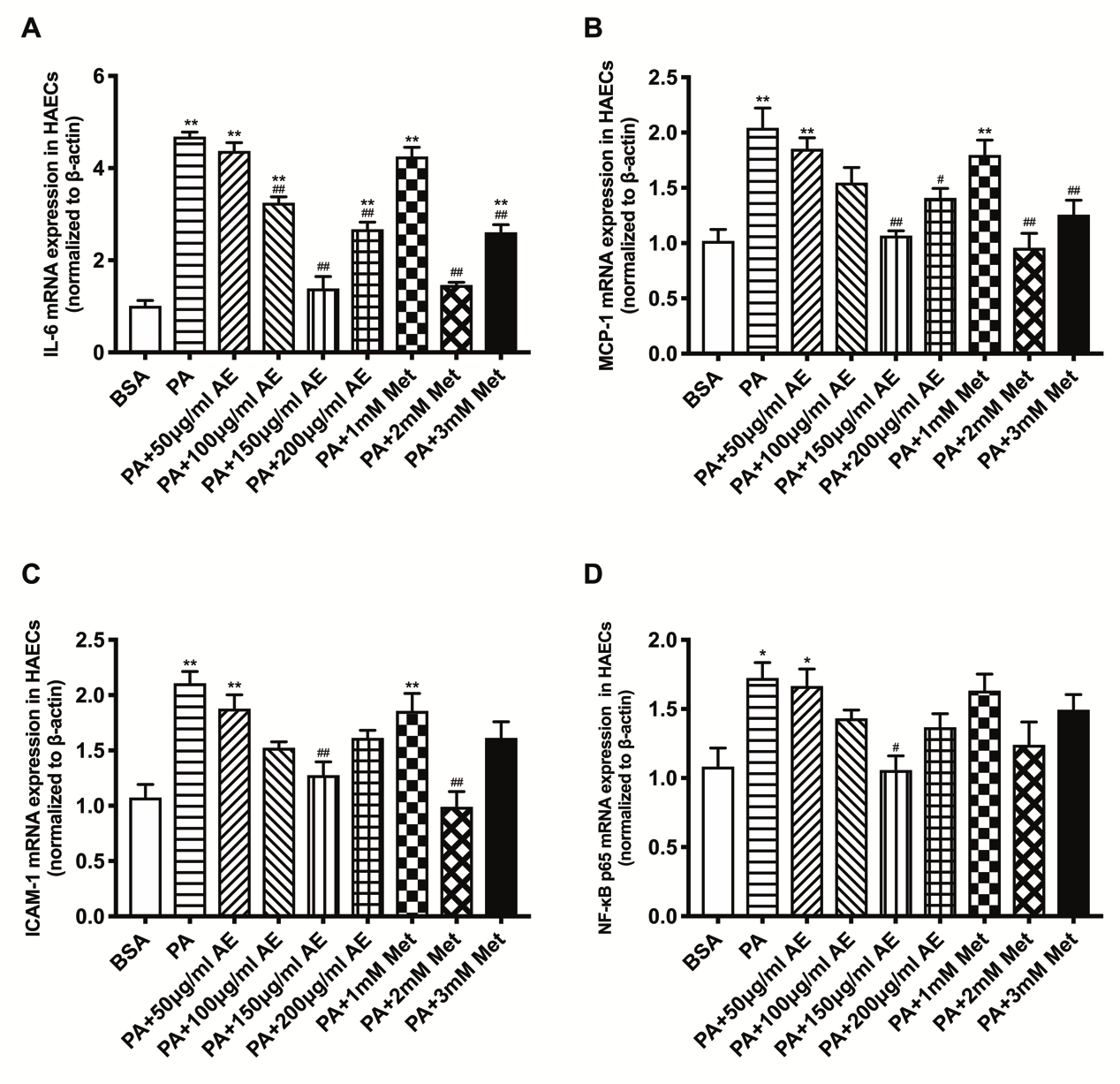


**Supplemental Figure 3.** **The effects of different concentrations of AE and metformin on the inflammation factors in HAECs after treatment with PA.** A, mRNA level of IL-6 in HAECs. B, mRNA level of MCP-1 in HAECs. C, mRNA level of ICAM-1 in HAECs. D, mRNA level of NF-κB p65 in HAECs. Data are presented as mean±SEM. ^*^*P* < 0.05 versus BSA group. ^**^*P* < 0.01 versus BSA group. ^#^*P*＜0.05 versus PA group. *^#^*^#^*P*＜0.01 versus PA group. n=3 per group for HAECs study.


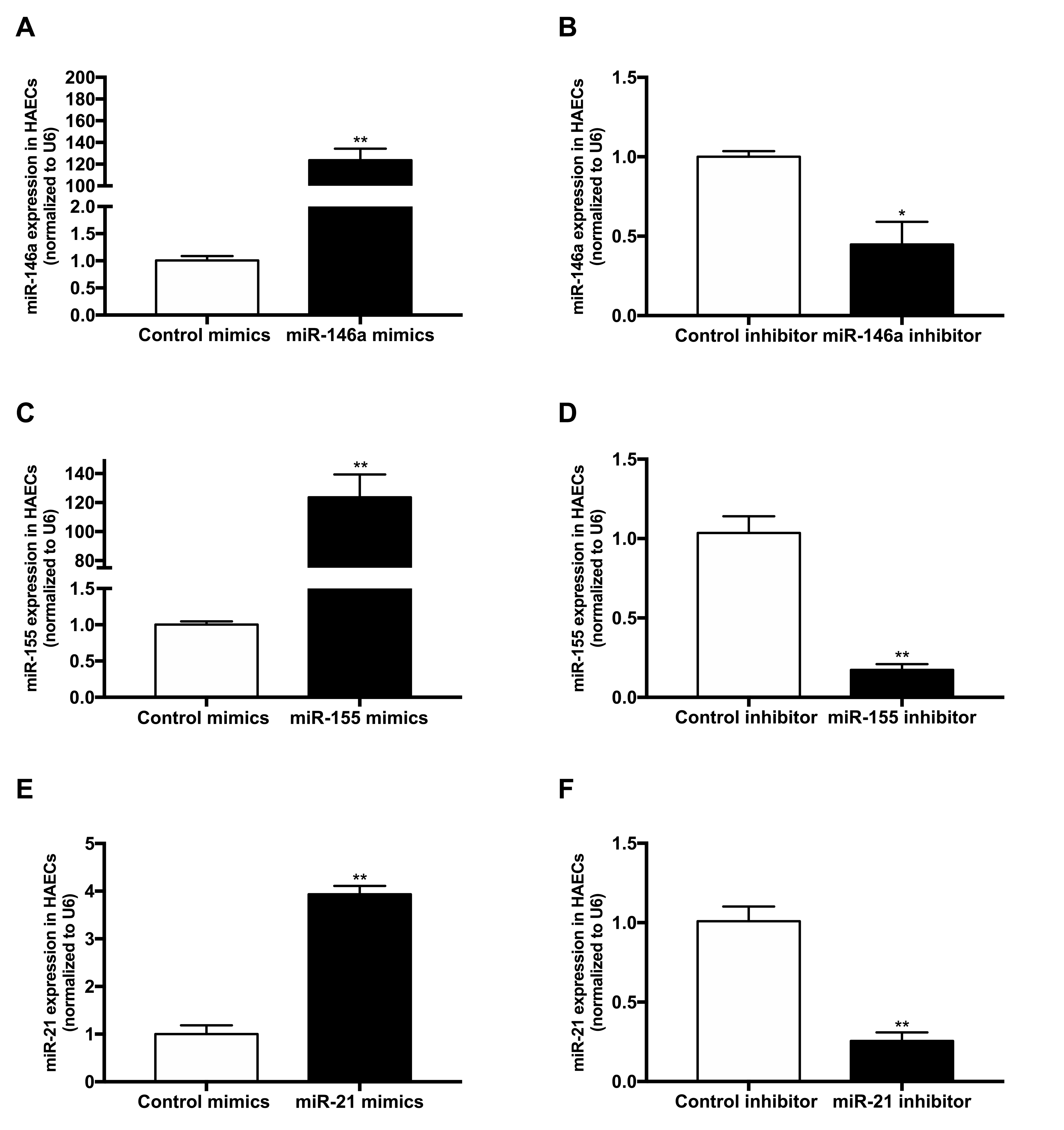


**Supplemental Figure 4. The relative expressions of miRNAs in HAECs after transfection with mimics or inhibitors.** A and B, miR-146a expression level in HAECs. C and D, miR-155 expression level in HAECs. E and F, miR-21 expression level in HAECs. Data are presented as mean±SEM. ^*^*P* < 0.05 versus control mimics or control inhibitors. ^**^*P* < 0.01 versus control mimics or control inhibitors. n=3 per group for HAECs study.

Supplemental Table 1. Primers for RT-PCR

| Gene | Forward primer, 5’-3’ | | Reverse primer, 5’-3’ |
| --- | --- | --- | --- |
| Human  NF-κB p65 | | ACCCCTTCCAAGAAGAGCAG | TCACTCGGCAGATCTTGAGC |
| Human  MCP-1 | | GAAAGTCTCTGCCGCCCTTC | GGTGACTGGGGCATTGATTG |
| Human IL-6 | | TGCAATAACCACCCCTGACC | ATTTGCCGAAGAGCCCTCAG |
| Human ICAM-1 | | TGTGACCAGCCCAAGTTGTT | AGTCCAGTACACGGTGAGGA |
| Human IRAK1 | | TCAGCTTTGGGGTGGTAGTG | TAGATCTGCATGGCGATGGG |
| Human TRAF6 | | GCACGCCACCTACAAGAGAA | CCCAGAGTCGGGTATAACGC |
| Human  β-actin | | TGGCACCCAGCACAATGAA | CTAAGTCATAGTCCGCCTAGAAGCA |
| Rat  NF-κB p65 | | TGTATTTCACGGGACCTGGC | CAGGCTAGGGTCAGCGTATG |
| Rat MCP-1 | | TGATCCCAATGAGTCGGCTG | GGTGCTGAAGTCCTTAGGGT |
| Rat IL-6 | | CTGGTCTTCTGGAGTTCCGTT | TGGAAGTTGGGGTAGGAAGGA |
| Rat ICAM-1 | | AGGGGAATCCAGCCCCTAAT | GCACGTCCCTGGTGATACTC |
| Rat IRAK1 | | GCGGGACATCATTACAGCCT | GGAGGGGGCTGAAGATTGTAG |
| Rat TRAF6 | | AGAGGAATCACTTGGCACGG | TCTGCGTTTCCATTTTGGCG |
| Rat β-actin | | CCTAAGGCCAACCGTGAAAA | GGTACGACCAGAGGCATACA |

Supplemental Table 2. The primary antibodies for Western Blotting

| Peptide/Protein target | Manufacturer (catalog number) | Species raised in; monoclonal or polyclonal | Dilution |
| --- | --- | --- | --- |
| p-NF-κB p65 | Cell Signaling Technology (3033) | Rabbit monoclonal | 1:1000 |
| NF-κB p65 | Cell Signaling Technology (8242) | Rabbit monoclonal | 1:1000 |
| IRAK1 | Proteintech (10478-2-AP) | Rabbit polyclonal | 1:1000 |
| TRAF6 | ABclonal (A0973) | Rabbit polyclonal | 1:1000 |
| β-actin | Cell Signaling Technology (4970) | Rabbit monoclonal | 1:1000 |
